# Interprofessional teamwork versus fast track streaming in an emergency department—An observational cohort study of two strategies for enhancing the throughput of orthopedic patients presenting limb injuries or back pain

**DOI:** 10.1371/journal.pone.0220011

**Published:** 2019-07-18

**Authors:** Jenny Liu, Italo Masiello, Sari Ponzer, Nasim Farrokhnia

**Affiliations:** Department of Clinical Science and Education, Södersjukhuset, Karolinska Institutet, Stockholm, Sweden; Medical University Graz, AUSTRIA

## Abstract

**Objective:**

To compare two strategies, interprofessional teams versus fast track streaming, for orthopedic patients with limb injuries or back pain, the most frequent orthopedic complaints in an emergency department.

**Methods:**

An observational before-and-after study at an adult emergency department from May 2012 to Nov 2015. Patients who arrived on weekdays from 8 am to 9 pm and presented limb injury or back pain during one year of each process were included, so that 11,573 orthopedic presentations were included in the fast track period and 10,978 in the teamwork period. Similarly, another 11,020 and 10,760 arrivals presenting the six most frequent non-orthopedic complaints were included in the respective periods, altogether 44,331 arrivals. The outcome measures were the time to physician (TTP) and length of stay (LOS). The LOS was adjusted for predictors, including imaging times, by using linear regression analysis.

**Results:**

The overall median TTP was shorter in the teamwork period, 76.3 min versus 121.0 min in the fast track period (-44.7 min, 95% confidence interval (CI): -47.3 to -42.6). The crude median LOS for orthopedic presentations was also shorter in the teamwork period, 217.0 min versus 230.0 min (-13.0 min, 95% CI: -18.0 to -8.0), and the adjusted LOS was 22.8 min shorter (95% CI: -26.9 to -18.7). For non-orthopedic presentations, the crude median LOS did not differ significantly between the periods (2.0 min, 95% CI: -3.0 to 7.0). However, the adjusted LOS was shorter in the teamwork period (-20.1 min, 95% CI: -24.6 to -15.7).

**Conclusions:**

The median TTP and LOS for orthopedic presentations were shorter in the teamwork period. For non-orthopedic presentations, the TTP and adjusted LOS were also shorter in the teamwork period. Therefore, interprofessional teamwork may be an alternative approach to improve the patient flow in emergency departments.

## Introduction

Crowding causes problems in emergency departments (ED) worldwide [1–3], such as a reduced quality of care, delayed pain care, prolonged in-hospital stays, increased mortality, and stressful work environments [4–6]. The causes of crowding are complex and vary between EDs [7], depending on input, throughput and output factors [8]. A common strategy used to improve the throughput of large EDs is to treat patients with minor complaints in a separate fast track process [1, 9]. Streaming these patients reduces the waiting time, ED length of stay (LOS) and the proportion of patients who leave without being seen by a physician [9].

Interprofessional teamwork, where different professions collaborate, is an alternative approach to improve ED throughput [10–12]. Teamwork improves patient safety [13–15], the quality of care, and the satisfaction of patients and staff [16, 17]. However, teamwork in ED settings has so far only been investigated in a small number of studies [10–12, 18–20]. As an example, we have previously reported that interprofessional teamwork reduces the overall ED LOS compared to triage led by nurses or physicians [12].

Patients presenting to an ED with limb injury or back pain make up nearly one third of the low acuity patients [21], and the ambulatory patients in this category often qualify for fast track streaming [22]. However, the non-ambulatory patients risk experiencing long waiting times and lengthy stays in crowded main EDs, where high acuity patients are given higher priority. To our knowledge, the ED flow of patients with these orthopedic complaints has not been studied, except for the case of hip fractures, where fast tracks have been designed to bypass the ED [23–26]. Although a number of authors have studied fast tracks streaming patients with minor complaints in EDs, only two studies specify the proportion of patients presenting limb injuries [21, 22], and none report the outcomes specifically for these patients.

The aim of this study is to compare the ED throughput of two different strategies for patients with limb injury or back pain, which are the most frequent orthopedic presentations. The ED throughput is measured in terms of the LOS and the waiting time to physician assessment (TTP). Our research questions are: Can interprofessional teams improve the ED throughput for all patients presenting limb injury or back pain, ambulatory as well as non-ambulatory, compared to a process with fast track streaming only for the ambulatory patients? And does this lead to longer times for patients with other surgical complaints?

## Materials and methods

### Study design and setting

We conducted an analytic observational cohort study with a before-and-after design during the time period from May 2012 to Nov 2015 at the adult ED of Södersjukhuset, a 600-bed level II trauma center with 110,000 ED visits per year located in central Stockholm, Sweden. Emergency medicine is a recent specialty in Sweden and this hospital was the first to introduce emergency physicians. While most Swedish EDs rely entirely on physicians from other departments who rotate for ED shifts, this ED has its own physicians who cover more than 50% of the shifts.

The one-year period from 2012.05.09 to 2013.05.08 was chosen as the control period of fast track streaming for ambulatory patients, and the first year after the implementation of interprofessional teamwork from 2014.11.12 to 2015.11.11 as the intervention period. There were several minor process changes in the interval between these periods, when three improvement groups carried out the activities that led to the teamwork intervention.

#### ED organization before the intervention

**Fig 1:**
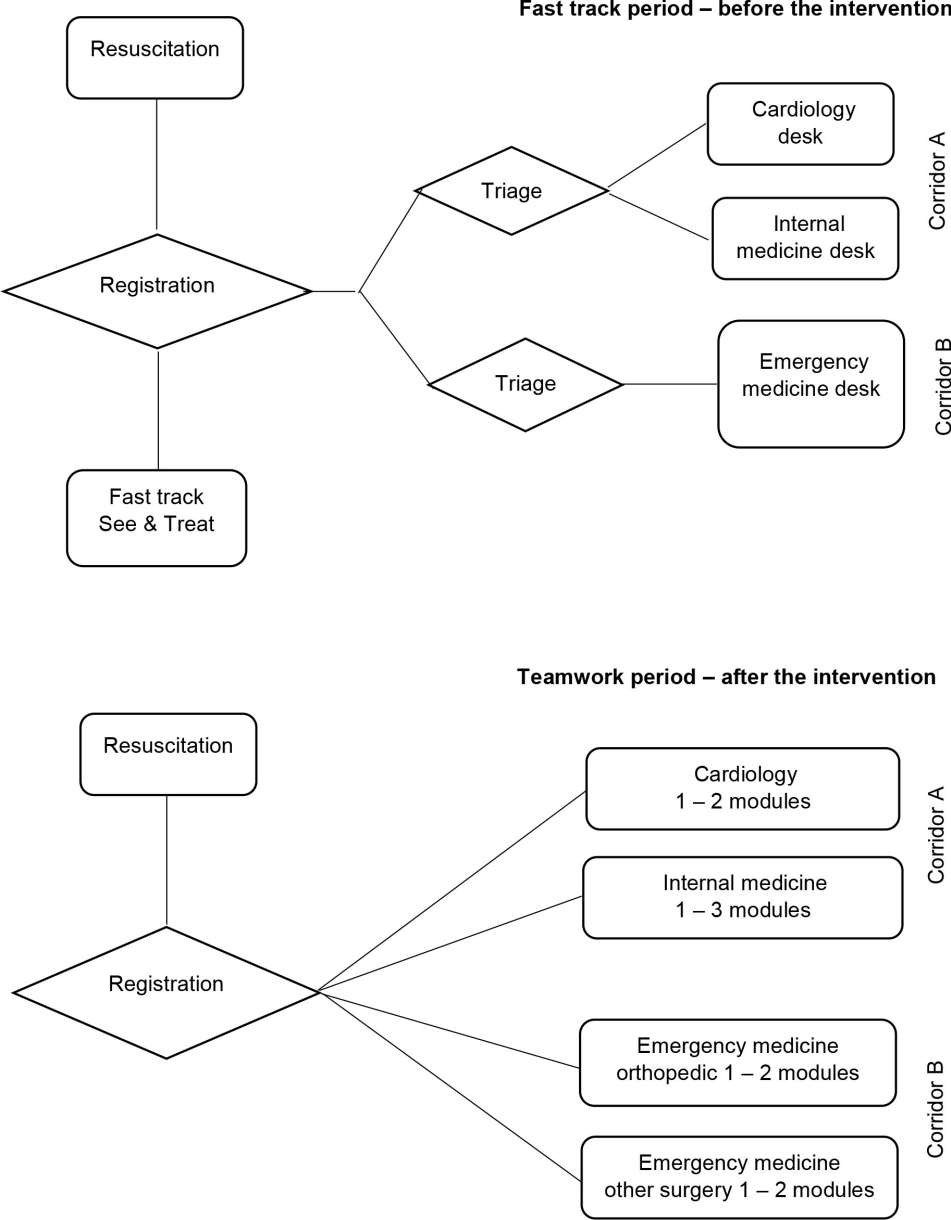
Emergency department organization. In both periods, physicians from the departments of internal medicine and cardiology treated their patient categories in corridor A. Patients with surgery complaints were treated in corridor B by physicians from the emergency department, who also staffed the fast track See & Treat until it was closed in the teamwork intervention.

The main ED was organized in two separate corridors. In corridor A, physicians from the departments of internal medicine and cardiology treated their respective patient categories, while patients with surgery complaints were treated in corridor B by physicians from the ED. In addition, one physician rotated from the orthopedic department daily from 8 am to 9 pm. All nursing staff belonged to the ED and rotated between all sections. (Fig 1)

A fast track called See & Treat was designed for ambulatory patients with minor complaints. It had a dedicated waiting area and eight rooms located 200 meters from the registration nurse, who directed suitable patients to See & Treat after a brief check. A physician was called to request radiographs for patients with limb injuries before they left the registration area. See & Treat operated daily from 8 am to 11 pm and was staffed by a senior and a junior physician, a nursing assistant, and a registered nurse. They were joined by an additional junior physician and a nursing assistant during peak hours from 11 am to 7 pm. All physicians and nursing staff belonged to the ED and rotated to See & Treat. On average, 18% (55/300 per day) of the ED patients were dispositioned from See & Treat, where the most frequent main complaints were: pain or swelling of extremity (25%), hand or arm injury (22%), abdominal pain (11%), foot injury (9%), knee or leg injury (6%), and low back pain (4%).

Non-ambulatory patients and patients with major complaints were directed by the registration nurse to the triage areas, where senior nurses conducted a comprehensive triage assessment based on the Rapid Emergency Triage and Treatment System (RETTS) [27, 28], before being transferred to a desk in the main ED. There, the next available doctor assessed the patient on his or her own and left written orders for the next available nurse to carry out. Each doctor shared patients with multiple nurses during a shift, and vice versa.

#### Interprofessional teamwork intervention

Interprofessional teamwork was implemented from 2014.11.12 on weekdays from 8 am to 9 pm. See & Treat was closed, and the main ED and the triage sections were re-organized into modules with dedicated rooms, bays, a waiting area, and a team area. Each module was staffed by a senior flow team and two care teams, where each team consisted of a doctor and a nurse. Doctors moved from their back offices to the team area and were placed next to their team nurse. The work schedules of the different professions were synchronized, which allowed team members to work an entire shift together.

**Fig 2:**
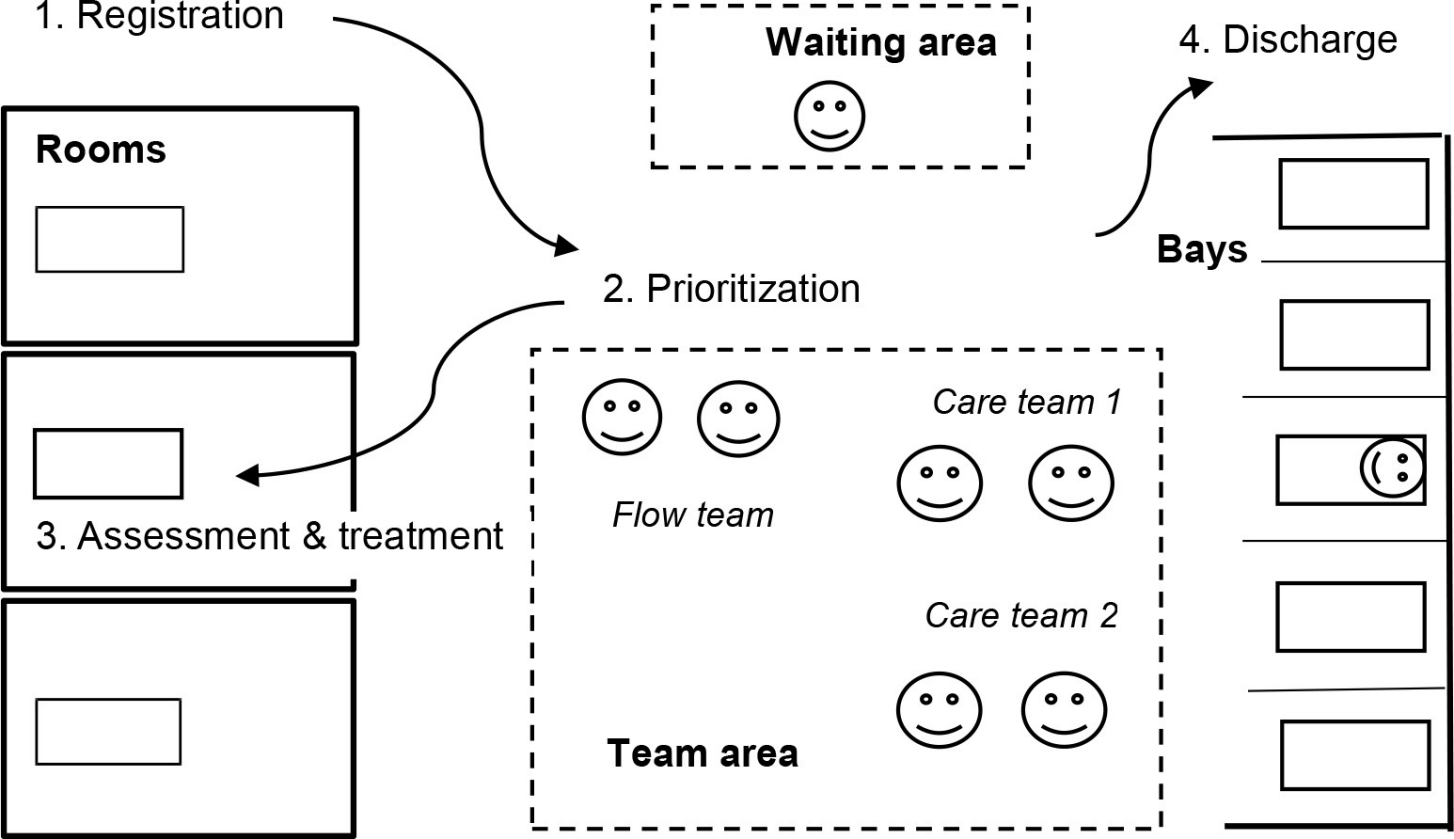
Interprofessional teamwork intervention. Depending on the main complaint, the patient was assigned to an appropriate teamwork module with dedicated rooms, bays, a waiting area, and a team area. The module was staffed by one senior flow team and two care teams, each consisting of a doctor and a nurse. The flow team nurse prioritized among the queuing patients, while each care team assessed and treated patients with support from the flow team doctor.

The patient flow in the teamwork period was structured in the following way: First, a registration nurse assigned the patient to an appropriate teamwork module according to his or her main complaint, internal medicine or cardiology in corridor A and orthopedic or other surgery complaints in corridor B. In the module, the flow team nurse prioritized and was responsible for the patient until assessment by a care team started. The doctor and nurse collaborated to carry out the patient interview, physical examination, radiology and laboratory orders, and in some cases treatment, in immediate sequence. In order to have time to lead and support the team in prioritizing and deciding the correct plans for the patients, the flow team doctor only treated low complexity patients. (Fig 2)

### Selection of participants and data collection

We extracted registry data of all adult visits to the ED during the fast track control period from 2012.05.09 to 2013.05.08, and the teamwork intervention period from 2014.11.12 to 2015.11.11 from the electronic tracking system Akusys. We extracted the following data: The time of each patient arrival, when the first physician signed in, and when the patient departed, as well as the main complaint, arrival mode, disposition, age, and gender of the patient. All patient identification numbers were replaced by unique codes.

There were three inclusion criteria for the study, which all had to be met. The first two criteria were: arrival on weekdays from 8 am to 9 pm, and a disposition from corridor B or See & Treat. The third was that the main complaint was one of the six most frequent orthopedic complaints: injury of shoulder, hand/arm, hip/thigh, knee/leg, feet, and back pain, or one of the six most frequent non-orthopedic complaints: head injury, abdominal pain, gastro-intestinal bleeding, flank pain, urinary problem, and genital complaint. Arrivals during night shifts, on weekends, and on holidays were excluded, since the teamwork intervention was not introduced during these work shifts. The primary outcome was the ED LOS, measured from the arrival registration to the departure. The secondary outcome was the TTP, measured from the arrival registration to the first physician sign-in.

In addition, we collected data from the electronic imaging registry. The time when an imaging request was made, when the examination started, and when a radiologist signed the result, as well as the type of imaging carried out were retrieved. From the work schedules, we collected the working hours for each profession in corridor B and See & Treat on weekdays from 8 am to 9 pm. Finally, we extracted the number of inpatients and beds per ward every weekday at 6 am from the electronic in-bed registry to calculate the daily occupancy rate for the wards which admitted patients from the adult ED.

### Statistical analysis

We imported the data retrieved from the ED tracking registry and the imaging registry into IBM SPSS Statistics version 25 for statistical analysis. We used the Chi-squared test to compare proportions and the Mann-Whitney-Wilcoxon test to compare mean values. The distributions of LOS, TTP and imaging times are heavily skewed with short times for most patients and a smaller number of very long times. Therefore, we used the median values for comparison. To obtain the 95% confidence intervals by bootstrap simulation, we also imported all retrieved data into R version 3.2.4 (The R Foundation for Statistical Computing, Vienna).

We performed a linear regression analysis to adjust the LOS for differences in background characteristics. First, we defined nine relevant predictors and explored each by simple linear regression before entering all predictors in a multiple linear regression model. Four of these were continuous predictors: age, daily ED volume, daily in-bed occupancy rate, and imaging turnaround time within ED stay. The remaining five were binary predictors: gender, study period, arrival by ambulance or helicopter without alert, arrival with prehospital alert, and one variable indicating if imaging was completed after the ED stay. We checked that the model assumptions were met using histograms, scatterplots, normal probability plots, and Cooke´s distance. First, we entered all included arrivals in the model and then several subgroups, based on disposition and main complaint. The statistical significance level was set at a two-tailed p-value of 0.05 for all outcomes.

### Ethics approval and consent

The study was approved by the Regional Ethical Review Board of Stockholm, ref. no. 2016/109-31/5. This included an approval of not obtaining informed consent of patients to participate in the study, since all patients were assessed and treated according the process during each period. We did not request consent for publication from the patients, since all data was anonymized and only reported at aggregated levels with many patients at each level.

## Results

### Characteristics of study subjects

**Fig 3:**
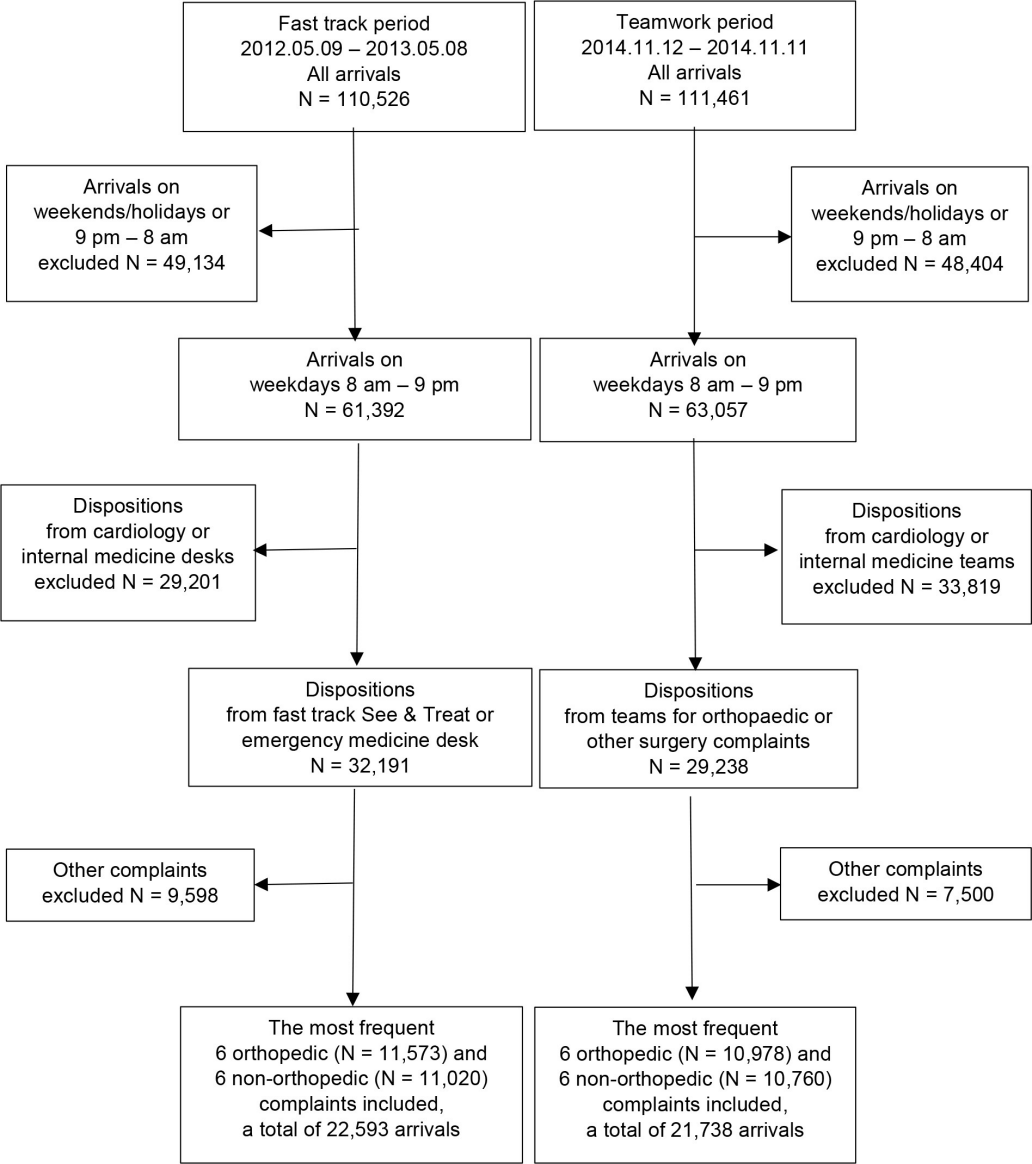
Flow diagram of the study population.

Out of a total of 110,526 patient arrivals during the fast track period from 2012.05.09 to 2013.05.08, we included 11,573 orthopedic presentations and another 11,020 non-orthopedic presentations on weekdays from 8 am to 9 pm. Similarly, from the teamwork period from 2014.11.12 to 2015.11.11, we included 10,978 orthopedic and another 10,760 non-orthopedic presentations, out of a total of 111,461 patient arrivals. Altogether, we included 44,331 patient arrivals in the study. (Fig 3)

Two general characteristics differed between the study periods. The patients included were older (mean age +1.8 years, 95% CI 1.4 to 2.2) in the teamwork period, when also a larger proportion of the non-orthopedic patients arrived by ambulance or helicopter (+1.7%, 95% CI: 0.5% to 2.8%). Moreover, the mean in-bed occupancy rate was higher in the teamwork period, 97.8% (SD 4.95%) compared to 92.6% (SD 4.84%) in the fast track period (+5.2%, 95% CI: 5.1% to 5.3%), due to a decrease in in-hospital beds and an increase in the number of patients admitted. In order to form interprofessional teams from 8 am to 9 pm in corridor B, more physician hours were scheduled in the teamwork period, 141.5 hours per weekday compared to 132.5 in the fast track period (+6.9%). However, the scheduled hours per weekday for the nursing staff was smaller in the teamwork period, 260.9 hours compared to 262.3 hours in the fast track period (-0.5%). (Table 1)

**Table 1:** General characteristics of the study population.

	Fast track period						Teamwork period		
	2012.05.09 - 2013.05.08						2014.11.12–2015.11.11		
	See & Treat		Main ED		Overall		Overall		
Age	N	Mean (SD)	N	Mean (SD)	N	Mean (SD)	N	Mean (SD)	*p*-value
Overall	8,392	40.6 (17.6)	14,201	56.1 (22.8)	22,593	50.4 (22.3)	21,738	52.2 (22.0)	<0.001
Orthopedic presentations	5,741	41.5 (17.8)	5,832	58.8 (23.2)	11,573	50.3 (22.5)	10,978	52.5 (22.0)	<0.001
Non-orthopedic presentations	2,651	38.6 (16.8)	8,369	54.3 (22.2)	11,020	50.5 (22.1)	10,760	52.0 (21.9)	<0.001
**Female gender**	**N**	**%**	**N**	**%**	**N**	**%**	**N**	**%**	
Overall	3,930	46.8	7,652	53.9	11,582	51.3	11,130	51.2	0.898
Orthopedic presentations	2,583	45.0	3,257	55.8	5,840	50.5	5,551	50.6	0.883
Non-orthopedic presentations	1,347	50.8	4,395	52.5	5,742	52.1	5,579	51.8	0.716
**Arrival by ambulance/helicopter; no alert**									
Overall	99	1.2	4,692	33.0	4,791	21.2	4,853	22.3	0.004
Orthopedic presentations	57	1.0	2,278	39.1	2,335	20.2	2,277	20.7	0.299
Non-orthopedic presentations	42	1.6	2,414	28.8	2,456	22.3	2,576	23.9	0.004
**Arrival with prehospital alert**									
Overall	0	0	410	2.9	410	1.8	412	1.9	0.529
Orthopedic presentations	0	0	98	1.7	98	0.8	90	0.8	0.824
Non-orthopedic presentations	0	0	312	3.7	312	2.8	322	3.0	0.479
**Low acuity–RETTS green or blue**									
Overall	8,226	98.0	4,036	28.4	12,262	54.3	NA	NA	
Orthopedic presentations	5,672	98.8	2,406	41.3	8,078	69.8	NA	NA	
Non-orthopedic presentations	2,554	96.3	1,630	19.5	4,184	38.0	NA	NA	<0.001^a^
**Staffing on weekdays 8 a.m. - 9 p.m.**					**N**		**N**		
Mean physician hours per day					132.5		141.5		
Mean hours for nursing staff per day					262.3		260.9		
**In-hospital beds on weekdays 6 a.m.**					**N**		**N**		
Mean number of in-patients					391		398		
Mean number of available beds					423		408		
					**%**	**(SD)**	**%**	**(SD)**	
Mean bed occupancy rate					92.6	(4.84)	97.8	(4.95)	<0.001

P-value <0.001^a^: difference in low-acuity patients between orthopedic and non-orthopedic presentations in the fast track period.

In the fast track period, 49.6% (N = 5,741) of the orthopedic presentations and 24.1% (N = 2,651) of the non-orthopedic presentations were dispositioned from See & Treat. These patients differed from those dispositioned from the main ED in several aspects. They were younger (mean age -15.5 years), more often male (odds ratio (OR) 1.33) and presented orthopedic complaints more often (OR 3.11). In addition, they rarely arrived by ambulance or helicopter (OR 0.02) and were seldom admitted (OR 0.09). While 98.0% (N = 8,226) at See & Treat were low acuity patients, i.e., green or blue based on RETTS, in the main ED only 28.4% (N = 4,036) were low acuity.

#### Imaging

The proportion of patients for whom computed tomography (CT) was requested was larger in the teamwork period, 18.9% (N = 4,113) compared to 16.8% (N = 3,805) in the fast track period (+2.1%, p<0.001). For patients who completed CT within their ED stay, the median time from arrival to a CT request was shorter in the teamwork period, 123.5 min compared to 135.2 min in the fast track period (-11.7 min, 95% CI: -17.4 to -5.2). However, the median time from the request to a result was longer in the teamwork period, 138.0 versus 111.7 min (+26.3 min, 95% CI: 21.8 to 30.4), largely due to longer waiting times for the CT to start (+22.0 min, 95% CI: 17.9 to 26.3).

On the other hand, other types of imaging, such as radiographs, were requested for a smaller proportion of the orthopedic presentations in the teamwork period, 51.4% (N = 5,642) versus 52.8% (N = 6,112) in the fast track period (-1.4%, p = 0.033). Moreover, these patients had a longer median time from arrival to an imaging request in the teamwork period, 83.4 min compared to 50.7 min in the fast track period (+32.7 min, 95% CI: 29.6 to 35.9). The median time from the request to the start of non-CT imaging remained unchanged at 24.0 min for both periods (Table 2).

**Table 2:** Imaging requested from the ED and imaging times for those completed within ED stay.

	**Fast track period**		**Teamwork period**		**Difference**	**p-value**	
***Imaging requested from the ED***	**N**	**%**	**N**	**%**	**%**		
**Patients with any imaging request**							
Overall	10,998	48.7	10,887	50.1	1.4	0.003	
Orthopedic presentations	6,673	57.7	6,292	57.3	-0.3	0.600	
Non-orthopedic presentations	4,325	39.2	4,595	42.7	3.5	<0.001	
**Patients with CT requests**							
Overall	3,805	16.8	4,113	18.9	2.1	<0.001	
Orthopedic presentations	561	4.8	648	5.9	1.1	<0.001	
Non-orthopedic presentations	3,244	29.4	3,465	32.2	2.8	<0.001	
**Patients with non-CT requests**							
Overall	7,190	31.8	6,771	31.1	-0.7	0.126	
Orthopedic presentations	6,112	52.8	5,642	51.4	-1.4	0.033	
Non-orthopedic presentations	1,078	9.8	1,129	10.5	0.7	0.082	
***Median time for patients completing imaging within ED stay***	**Fast track period**		**Teamwork period**		**Difference**	**95% CI**	
	**N**	**min**	**N**	**min**	**min**	**Lower**	**Upper**
**CT—From arrival to request**							
Overall	2,755	135.2	2,888	123.5	-11.7	-17.4	-5.2
Orthopedic presentations	476	123.8	542	119.1	-4.7	-22.3	16.3
Non-orthopedic presentations	2,279	136.9	2,346	124.0	-12.9	-18.9	-6.5
**CT—From request to start of imaging**							
Overall	2,754	61.0	2,888	83.0	22.0	17.9	26.3
Orthopedic presentations	475	58.0	542	75.5	17.5	6.5	25.0
Non-orthopedic presentations	2,279	61.0	2,346	84.0	23.0	18.1	27.0
**CT—From request to result**							
Overall	2,755	111.7	2,888	138.0	26.3	21.8	30.4
Orthopedic presentations	476	113.5	542	139.0	25.5	16.0	36.0
Non-orthopedic presentations	2,279	111.0	2,346	138.0	27.0	22.0	31.5
**Non-CT—From arrival to request**							
Overall	6,156	55.2	5,698	86.1	30.9	27.7	34.1
Orthopedic presentations	5,707	50.7	5,221	83.4	32.7	29.6	35.9
Non-orthopedic presentations	449	140.0	477	121.9	-18.1	-31.8	0.5
**Non-CT—From request to start of imaging**							
Overall	6,148	24.0	5,697	24.0	0.0	-2.0	2.0
Orthopedic presentations	5,701	22.0	5,221	22.0	0.0	-2.0	1.0
Non-orthopedic presentations	447	56.7	476	49.0	-7.7	-17.2	-1.8
**Non-CT—From request to result**							
Overall	6,156	55.0	5,697	58.0	3.0	2.0	5.0
Orthopedic presentations	5,707	53.0	5,221	56.0	3.0	1.0	5.0
Non-orthopedic presentations	449	86.3	476	81.0	-5.3	-15.2	3.8

Abbreviations: CI = Confidence interval. CT = computed tomography. ED = emergency department

### Main results

#### Time to physician

In the teamwork period, the median TTP was considerably shorter for all main complaints compared to the fast track period. The median TTP was 70.0 min for orthopedic presentations and 84.4 min for non-orthopedic presentations in the teamwork period, compared to 127.0 min and 114.0 min, respectively, in the fast track period. This means that the reduction in median TTP was greater for orthopedic presentations, -57.0 min (95% CI: -60.1 to -53.9) versus -29.6 min (95% CI: -33.2 to -26.2) for non-orthopedic presentations. In fact, the longer median TTP found for orthopedic presentations in the fast track period (+13.0 min, 95% CI: 9.0 to 17.0), turned out to be shorter in the teamwork period (-14.4 min, 95% CI: -17.2 to -11.4) when compared to non-orthopedic presentations. (Table 3)

**Table 3:** Median time to physician and median length of stay per main complaint.

	Fast track period						Teamwork period		Difference		
	See & Treat		Main ED		Overall		Overall		Overall	95% CI	
	N	min	N	min	N	min	N	min	min	Lower	Upper
**Time to physician**											
Overall	8,078	136.0	13,665	107.0	21,743	121.0	21,188	76.3	-44.7	-47.3	-42.6
Orthopedic complaints	**5,483**	**136.0**	**5,610**	**111.0**	**11,093**	**127.0**	**10,717**	**70.0**	**-57.0**	**-60.1**	**-53.9**
Shoulder injury	302	150.5	495	86.0	797	111.0	823	63.5	-47.5	-56.8	-37.1
Hand/arm injury	2,761	134.0	1,204	113.0	4,132	129.0	3,754	64.3	-64.7	-69.4	-58.9
Hip/thigh injury	69	135.0	1,223	87.5	1,273	90.0	1,299	66.4	-23.6	-30.6	-15.1
Knee/leg injury	768	142.5	709	139.0	1,477	142.0	1,435	77.9	-64.1	-73.4	-57.7
Foot injury	1,092	135.5	682	114.0	1,774	130.0	1,704	58.4	-71.6	-78.4	-65.4
Back pain	491	141.0	1 149	140.0	1 640	140.5	1 702	99.6	-40.9	-51.3	-32.2
Non-orthopedic complaints	**2,595**	**135.0**	**8,055**	**103.0**	**10,650**	**114.0**	**10,471**	**84.4**	**-29.6**	**-33.2**	**-26.2**
Head injury	372	139.5	1,867	85.0	2,239	96.0	2,441	69.3	-26.7	-31.6	-20.3
Abdominal pain	1,449	136.0	4,326	112.0	5,775	121.0	5,606	92.6	-28.4	-33.0	-23.8
GI bleeding	36	160.0	369	72.0	405	76.0	276	54.4	-21.6	-44.7	-10.6
Flank pain	100	132.5	456	102.0	556	111.0	769	90.6	-20.4	-32.8	-6.7
Urinary	267	131.0	655	132.0	922	132.0	697	100.1	-32.0	-41.3	-20.9
Genital	371	130.0	382	94.0	753	111.0	682	66.1	-44.9	-57.1	-32.9
**LOS all dispositions**											
Overall	8,392	194.0	14,201	292.0	22,593	244.0	21,738	241.0	-3.0	-7.0	1.0
Orthopedic complaints	**5,741**	**191.0**	**5,832**	**295.0**	**11,573**	**230.0**	**10,978**	**217.0**	**-13.0**	**-18.0**	**-8.0**
Shoulder injury	312	203.5	510	267.0	822	236.0	835	226.0	-10.0	-24.0	7.0
Hand/arm injury	2,881	191.0	441	280.0	4,322	210.0	3,859	180.0	-30.0	-36.0	-23.0
Hip/thigh injury	74	206.0	1,223	314.0	1,297	306.0	1,313	315.0	9.0	-3.0	22.0
Knee/leg injury	806	201.0	748	289.0	1,554	231.0	1,469	224.0	-7.0	-20.5	4.0
Foot injury	1,150	178.0	726	253.5	1,876	201.0	1,751	177.0	-24.0	-33.0	-15.0
Back pain	518	188.5	1,184	330.5	1,702	274.0	1,751	276.0	2.0	-13.0	16.0
Non-orthopedic complaints	**2,651**	**201.0**	**8,369**	**289.0**	**11,020**	**261.0**	**10,760**	**263.0**	**2.0**	**-3.0**	**7.0**
Head injury	387	180.0	1,966	298.0	2,353	272.0	2,531	290.0	18.0	4.0	31.0
Abdominal pain	1,474	212.0	4,477	300.0	5,951	272.0	5,731	268.0	-4.0	-10.0	4.0
GI-bleeding	36	198.0	378	229.0	414	222.5	281	229.5	7.0	-13.0	31.5
Flank pain	103	217.0	465	260.0	568	249.0	791	252.0	3.0	-12.0	20.0
Urinary	270	182.5	685	284.0	955	251.0	720	249.0	-2.0	-23.0	13.0
Genital	381	184.0	398	217.0	779	198.0	706	172.0	-26.0	-44.5	-13.5
**LOS discharged home**											
Overall	7,744	191	7,825	275	15,569	223.0	14,427	209.0	-14.0	-17.0	-10.0
Orthopedic complaints	**5,490**	**190**	**3,751**	**272**	**9,061**	**214.0**	**8,414**	**186.0**	**-28.0**	**-33.0**	**-24.0**
Non-orthopedic complaints	**2,254**	**192**	**4,254**	**277**	**6,508**	**237.0**	**6,013**	**241.0**	**4.0**	**-2.0**	**9.5**
**LOS admitted**											
Overall	437	265	5,206	306	5,643	303.0	5,866	298.5	-4.5	-13.0	2.0
Orthopedic complaints	**93**	**293**	**1,682**	**312**	**1,775**	**311.0**	**1,795**	**320.0**	**9.0**	**-3.0**	**21.0**
Non-orthopedic complaints	**344**	**261.5**	**3,524**	**304**	**3,868**	**299.0**	**4,071**	**288.0**	**-11.0**	**-17.0**	**-1.0**

Abbreviations: CI = Confidence interval. GI = Gastro-intestinal. LOS = length of stay

#### Length of stay

The median LOS for the orthopedic presentations was shorter in the teamwork period, 217.0 min compared to 230.0 min in the fast track period (-13.0 min, 95% CI: -18.0 to -8.0). At the same time, the median LOS did not change significantly for the non-orthopedic presentations (+2.0 min, 95% CI: -3.0 to 7.0). We also compared the subgroup of orthopedic patients who were discharged home without imaging, since they were less affected by changes in imaging times and in-bed occupancy rate between periods. For these patients, the median LOS was shorter in the teamwork period, 143.0 min (N = 3,977) versus 180.0 min (N = 4,180) in the fast track period (-37.0 min, 95% CI: -42.0 to -29.0). In the fast track period, the median LOS was shorter for patients dispositioned from See & Treat compared to those dispositioned from the main ED (-98.0 min, 95% CI: -104.0 to -94.0), despite a longer median TTP (+29.0 min, 95% CI: 25.0 to 32.0) (Table 3).

In the multiple linear regression analysis, the adjusted LOS was shorter in the teamwork period for all main complaints, except gastro-intestinal bleeding and flank pain. The reduction of LOS in the teamwork period was approximately equal for orthopedic presentations (-22.8 min, 95% CI: -26.9 to -18.7) and non-orthopedic presentations (-20.1 min, 95% CI: -24.6 to -15.7). However, it varied depending on patient disposition. For orthopedic presentations, the reduction of LOS was larger for those discharged home (-28.5 min, 95% CI: -32.7 to -24.2), compared to those admitted (-22.5 min, 95% CI: -33.5 to -11.5). On the other hand, among non-orthopedic presentations the LOS reduction was larger for the admitted patients (-32.4 min, 95% CI -39.8 to -25.0), than for those discharged home (-14.9 min, 95% CI: -20.5 to -9.4). (Table 4)

**Table 4:** Multiple linear regression analysis with ED length of stay as dependent variable.

Overall model using all included patients Unstandardized B of all predictors specified		95% CI	
	B	Lower	Upper
Constant (min)	-189	-217	-160
Age (year)	0.869	0.802	0.936
Female gender (Yes = 1. No = 0)	20.2	17.5	22.9
Arrival by ambulance/helicopter without alert (Yes = 1. No = 0)	40.7	37.1	44.3
Arrival with prehospital alert (Yes = 1. No = 0)	-67.8	-77.8	-57.8
Imaging completed after discharge (Yes = 1. No = 0)	28.1	23.6	32.7
Time from request to result (min) for imaging completed within ED stay	1.09	1.06	1.11
Daily total volume (Range from 190 to 331 arrivals)	0.650	0.595	0.705
In-bed occupancy rate (Range from 0.719 to 1.091)	214	187	242
Teamwork period (Yes = 1. No = 0)	-21.0	-24.1	-18.0
**Model using patients per complaint** **Only B for predictor Teamwork period specified**			
Orthopedic presentations—all dispositions	**-22.8**	**-26.9**	**-18.7**
Shoulder injury	-21.6	-34.4	-8.7
Hand/arm injury	-27.3	-33.6	-21.1
Hip/thigh injury	-17.5	-29.4	-5.6
Knee/leg injury	-22.5	-33.8	-11.3
Foot injury	-27.3	-36.3	-18.4
Back pain	-22.6	-34.5	-10.7
Non-orthopedic presentations—all dispositions	**-20.1**	**-24.6**	**-15.7**
Head injury	-21.8	-31.1	-12.6
Abdominal pain	-16.9	-23.0	-10.8
GI bleeding	10.9	-14.4	36.3
Flank pain	-14.6	-30.6	1.5
Urinary	-29.9	-46.4	-13.4
Genital	-28.5	-42.8	-14.3
**Model using only patients discharged home** **Only B of predictor Teamwork period specified**			
Orthopedic presentations	**-28.5**	**-32.7**	**-24.2**
Non-orthopedic presentations	**-14.9**	**-20.5**	**-9.4**
**Same model using only admitted patients** **Only B of predictor Teamwork period specified**			
Orthopedic presentations	**-22.5**	**-33.5**	**-11.5**
Non-orthopedic presentations	**-32.4**	**-39.8**	**-25.0**

Abbreviations: B = Beta coefficient. CI = Confidence interval. ED = Emergency department

## Discussion

This study evaluated the ED throughput of two strategies for patients presenting common orthopedic complaints. We found that the adjusted LOS was reduced by 23 min when interprofessional teams treated all patients, ambulatory and non-ambulatory, compared to a process where the ambulatory patients were streamed in a fast track. The adjusted LOS was also reduced, by 20 min, for patients with non-orthopedic complaints. Moreover, we found a substantial reduction of the median TTP by 57 min for orthopedic presentations, and 30 min for non-orthopedic presentations.

One could argue that these results were caused by abolishing the comprehensive triage based on the RETTS protocol. However, the LOS was also reduced for typical See & Treat patients, such as those discharged home after upper limb injuries. These patients would have by-passed the RETTS triage in the fast track period. One reason for the decreased LOS in this group could be the role of the flow team doctor, who treated only low complexity patients, and in that way served as a fast track within the teamwork module. Another reason could be that professionals with orthopedic competencies, such as the cast technician and orthopedic surgeon, worked together in the same teamwork module, instead of being consulted from the main ED and See & Treat.

A direct comparison between periods for fast track patients was not possible, since See & Treat was closed in the teamwork period. However, an approximate comparison can be made for a subgroup that in the control period were highly likely to be streamed as fast track patients. For example, of 3,646 patients with hand or arm injury who did not arrive by ambulance or helicopter and were discharged home, 2,734 were dispositioned from See & Treat (OR 3.0). For these patients, the median LOS was considerably shorter in the teamwork period, 168.0 min (N = 3,268) versus 192.0 min in the fast track period.

Fast tracks vary in many aspects, such as operating hours, provider and patient categories. Compared to other fast tracks with similar operating hours and providers, such as those studied in [22, 29–32], the median TTP was longer for the fast track patients in this study. This may be due to the 200-meter distance from the ED registration to See & Treat, and the larger proportion of fast track patients in this study. Moreover, a comprehensive process was carried out in the ED for patients with fractures, which included immobilization, repeat radiographs, decision for surgery, and follow-up scheduling. In some EDs, such patients are instead referred to the fracture clinic [22].

The large cohort and long study periods of this investigation are strengths rarely found in similar studies. In addition, we adjusted the LOS for in-bed occupancy and imaging times. A 15.5 min yearly increase of the ED LOS for admitted patients, and 7.7 min per year for discharged patients has been reported in Sweden for the period from 2009 to 2016 [33]. Nevertheless, the adjusted LOS for admitted patients in our study was considerably shorter in the later teamwork period, -23 min for orthopedic presentations and -32 min for non-orthopedic presentations.

The use of advanced imaging for ED patients has been increasing globally over the last two decades [34–37]. This increase was also seen at the ED of this study, which together with a shortage of staff at the radiology department contributed to longer imaging times in the teamwork period. For non-CT imaging, the median time from patient arrival to a request was shorter in the fast track period, when radiographs for limb injuries were requested directly after registration. Nevertheless, the median TTP and LOS was shorter in the teamwork period, and a smaller proportion of the orthopedic presentations had radiographs. This indicates that a physician assessment in the teamwork module may reduce LOS and the need of radiographs, compared to when radiographs were requested directly after registration.

### Limitations

We are aware that the observational pre- and post-intervention design may not claim a causality between the teamwork intervention and the outcomes, although we have chosen study periods without other process changes and adjusted for potential sources of bias. However, the 6.9% increase of physician hours was not entered as an independent variable in the regression model due to collinearity in the dataset, which means that its contribution to a shorter TTP and LOS in the teamwork period could not be determined. In addition, two arrival modes were entered as predictors of case mix instead of standard triage categories, which were assigned systematically only in the fast track period. Finally, this study was conducted at the largest ED in Sweden, so our results may not be generalizable or transferrable to other settings.

## Conclusions

A process where patients with limb injuries and back pain, ambulatory and non-ambulatory, were treated by interprofessional teams in the ED, resulted in a shorter median TTP and LOS compared to a process with fast track streaming only for the ambulatory patients. The TTP and adjusted LOS for non-orthopedic patients was also reduced. This suggests that an improved ED patient flow may be an additional benefit of interprofessional teamwork in health care. However, these findings need to be confirmed by further studies, preferably with randomized designs and in other settings.
